# Adherence of Doctors to Standard Diarrhoeal Management Guideline During Treatment of Under-Five Diarrhoeal Episodes: A Study From Eastern India

**DOI:** 10.7759/cureus.13433

**Published:** 2021-02-18

**Authors:** Priyamadhaba Behera, Vikas Bhatia, Dinesh P Sahu, Durgesh Prasad Sahoo, Raviraj Kamble, Prem Panda, Arvind K Singh

**Affiliations:** 1 Community Medicine and Family Medicine, All India Institute of Medical Sciences, Bhubaneswar, Bhubaneswar, IND; 2 Community Medicine and Family Medicine, All India Institute of Medical Sciences, Bibinagar, Hyderabad, IND

**Keywords:** under-five diarrhoea, prescription audit, tribal area, india

## Abstract

Introduction

Diarrhoea is one of the major preventable causes of childhood death in tribal areas of India. Most acute diarrhoea in childhood can be managed with oral rehydration salt (ORS) and zinc. This study aimed to assess the adherence of doctors to standard diarrheal management guidelines while treating under-five diarrhoeal episodes.

Methods

The cross-sectional study was conducted in 10 blocks of Kandhamal district in southern Odisha, India. The under-five childhood diarrhoea prescriptions from July to August 2018 were audited during September 2018. One health facility from each block and 15 prescriptions from each health facility were selected randomly. Data were collected and entered in Epicollect5 and analyzed using Statistical Packages for Social Sciences Version 22.0 (IBM Corp., Armonk, NY). Categorical variables were presented as proportions.

Results

A total of 150 under-five acute diarrhoea prescriptions were audited from 10 health facilities. One hundred ten prescriptions were from the out-patient department and 40 prescriptions were from the admitted diarrhoeal patients. The majority of them included ORS (77.3%) and zinc (75.3%) in the prescription, however, only half of the prescriptions (52.7%) had recommended dose and duration of zinc. All admitted patients received intravenous fluids. Most prescriptions (89.3%) did not document the hydration status of the patient. All prescriptions were silent about the severe acute malnutrition status of the children before administering fluid therapy. Antibiotics were prescribed in 80% of the prescriptions. Prebiotics, probiotics and anti-spasmodic were prescribed in 37.3% of the prescriptions.

Conclusion

Adherence of doctors to acute diarrheal management guidelines for the management of under-five diarrhoea was poor in our study. Further researches and training are required to improve childhood diarrhoea management in health facilities of tribal areas of India.

## Introduction

Diarrhoea is the second leading cause of death among under-five year children claiming around 525,000 deaths every year [1]. World Health Organization (WHO) has recommended evidence-based cost-effectiveness strategies to reduce the burden of diarrheal disease. The strategies include oral rehydration solution (ORS), zinc supplementation and a nutritious diet. However, antibiotics are not required for most diarrheal conditions. It is only recommended for septicaemic illnesses, persistent diarrhoea or dysentery [2]. The under-five year children are treated either at home or health facilities depending on the severity and dehydration status [3]. Proper implementation of the diarrhoeal management guidelines improves child survival. But, all these interventions are not effectively implemented [4].

In tribal areas, diarrhoea is one of the most important causes of morbidity and mortality among under-five children due to the lack of safe drinking water, inaccessibility to the health facility, unhygienic condition and lack of awareness [5]. Giri et al. found a prevalence of 5.8% for childhood morbidity patterns in a tribal area of Maharastra [6]. The study by Mohapatra et al. from the tribal area of Odisha reported the lack of availability of anti-diarrhoeal medicines at the village level, lack of proper sanitation and lack of awareness were the prime reasons for under-five diarrhoea incidence and mortality [7]. Poor adherence to standard diarrhoeal management guidelines was observed in India [8]. In tribal areas where there is poor knowledge of the people regarding diarrhoea, adherence to diarrheal management guidelines is the key to tackle the mortality associated with diarrhoeal diseases. This study aimed to assess the adherence of doctors to standard diarrheal management guidelines while treating under-five diarrhoeal episodes.

## Materials and methods

**Figure 1:**
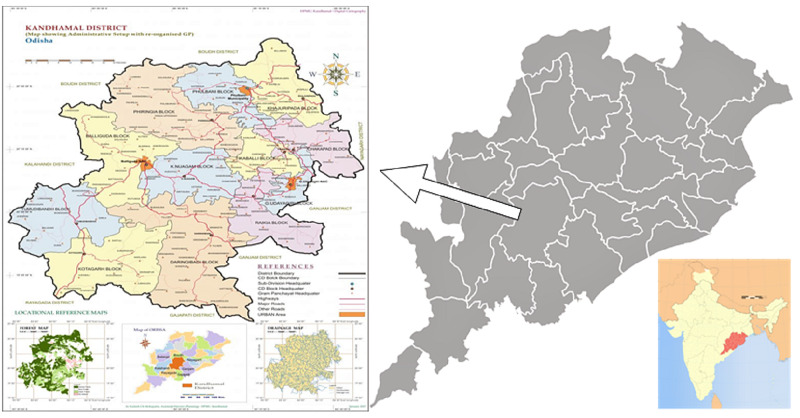
Map of the study area

**Figure 2:**
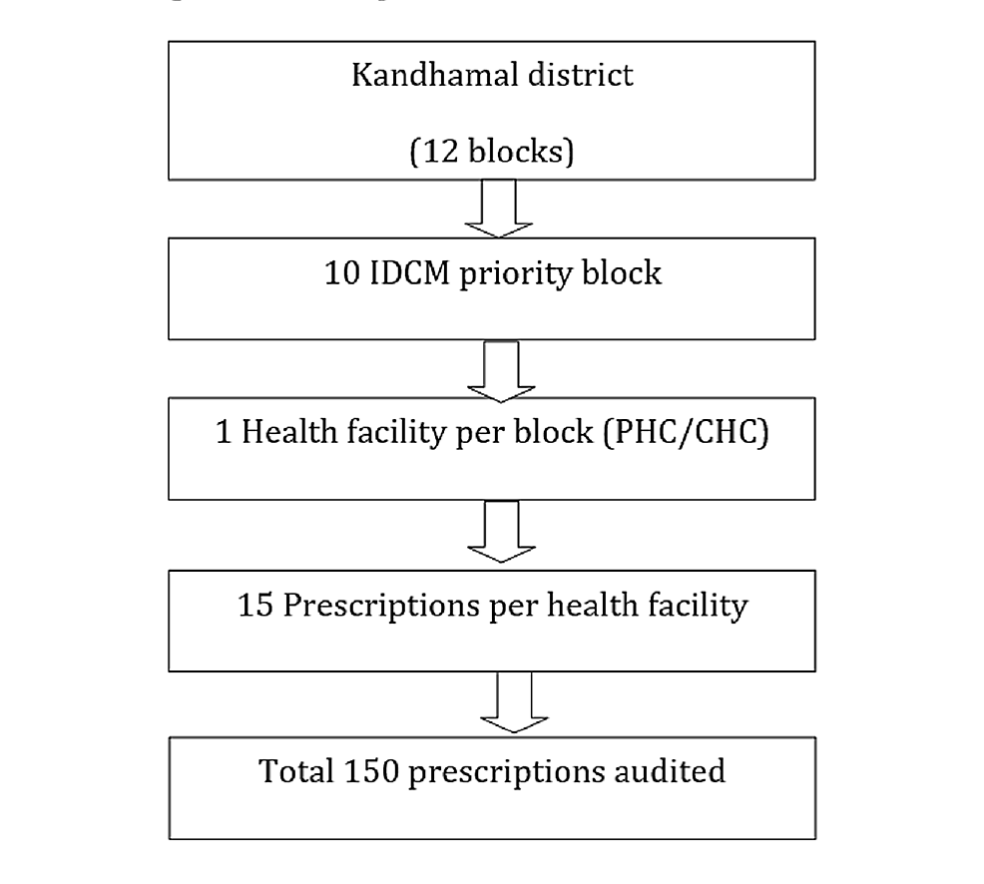
Flow of participants

Aspirational districts are the high priority districts identified based on poverty, poor health and nutrition, education status and deficient infrastructure. Kandhamal is one of the 115 identified aspirational districts [9]. The cross-sectional survey was conducted in a tribal predominant district of Odisha, Kandhamal during the month of September 2018. This aspirational district caters to around 0.7 million population and is divided into 12 administrative blocks (Figure 1). Out of the 12 blocks, 10 are priority blocks for intensified diarrhoea control month (IDCM) based on the diarrheal burden. The district health administration provided the list of blocks having a high burden of diarrhoea during the previous year. Assuming 50% of the prescriptions were correctly prescribed, absolute precision of 8% and desired confidence level of 95%, the sample size was estimated to be 150. One health facility from each block was selected randomly for a prescription audit. The prescription audit was conducted in September 2018. The prescriptions of under-five diarrhoea from July to August 2018 were considered for the audit. All the prescriptions of 10 selected health facilities, having a diagnosis of diarrhoea in the last two months, were line-listed. From each health facility, 15 prescriptions of under-five children having diarrhoea were selected randomly and audited. A total of 150 under five diarrhoea prescriptions were audited from 10 health facilities (Figure 2). Prescriptions were checked for completeness, accuracy and adherence to standard diarrhoeal disease management guidelines. This study was conducted by the Community Medicine department of All India Institute of Medical Sciences, Bhubaneswar. The prescription audit was conducted by four specialists of community medicine from All India Institute of Medical Sciences, Bhubaneswar.

Diarrhoea is defined by the WHO as a loose stool for three or more times in 24 hours [10]. According to the dehydration status, diarrhoea is classified into three categories, namely diarrhoea without dehydration, diarrhoea with some dehydration and diarrhoea with severe dehydration. For the management of diarrhoea, plans A, B and C were proposed by the world health organization. It suggests that plan A has to be adopted if there was no sign of dehydration or fluid deficit as <5% of the body weight, plan B for some dehydration or fluid deficit as 5-10% of the body weight and plan C for severe dehydration or fluid deficit as >10% of the body weight. Plan A to be managed at home, plans B and C have to be managed at the hospital. Plan C is again divided into C1 and C2, where C2 is specific for malnutrition children having diarrhoea. Therefore, the assessment of malnutrition is also essential before administering fluid therapy. Zinc is recommended with a dose of 20 mg for 14 days for children of six months to five years to reduce the frequency and severity of diarrhoea, and for children, less than six months is 10 mg for 14 days. The components of the diarrheal prescription also include the mention of the classification of diarrhoea after proper clinical examination. Antibiotics are recommended only for cases with gross blood in stools, cholera, associated systemic infection, or severe malnutrition [11]. Presently, there is insufficient evidence to recommend probiotics and anti-secretory drugs for the treatment of diarrhoea [12].

Data were collected and entered into Epicollect5 software and were extracted in an excel sheet. The analysis was done using a statistical package for social sciences version 22. All the categorical data were presented in percentage or proportion. Ethical approval was obtained from the institute ethics committee (T/IM-NF/CM&FM/18/39) of All India Institute of Medical Sciences, Bhubaneswar, and permission was obtained from the chief district medical officer, Kandhamal, Odisha.

## Results

The prescription audit was done for 150 prescriptions from the 10 health facilities (one from each block). One hundred ten prescriptions were from the out-patient department and 40 prescriptions were from the admitted diarrhoeal patients. Out of the 150 prescriptions, antibiotics were prescribed in 120 (80%) of the prescriptions, ORS was prescribed in 116 (77.3%), zinc was prescribed in 113 (75.3%), 40 admitted patients (26.7%) received intravenous fluids and prebiotics, probiotics and anti-spasmodic were prescribed in 56 (37.3%) prescriptions (Table 1). Thirty-seven (24.7%) under-five diarrhoea prescriptions did not contain zinc. Out of 113 prescriptions, 79 (52.7%) prescriptions were accurate for recommended dose and duration of zinc, the recommended duration of zinc was wrong in 14 (9.3%) prescriptions, the recommended dose of zinc was wrong in 11 (7.3%) prescriptions, both the recommended dose and duration of zinc was wrong in 9 (6.0%) prescriptions (Table 2). Most prescriptions - 137(89.3%) - were silent about the dehydration status of the patient. Out of 150 prescriptions, only 16 (11.7%) prescriptions contain information about dehydration status, and 12 prescriptions classified dehydration the status as per guideline. One prescription (0.7%) classified dehydration as severe dehydration, five (3.3%) prescriptions classified dehydration as some dehydration and four (2.7%) prescriptions classified dehydration as no dehydration (Table 3). All (150) prescriptions were silent about the severe acute malnutrition status of under-five children before administrating fluid therapy.

**Table 1:** Medicine prescribed for the management of under-five diarrhoea by the doctors (N=150)

Medicine prescribed for the management of diarrhoea by doctors	N (%)
Antibiotics	120 (80.0)
ORS prescribed	116 (77.3)
Zinc prescribed	113 (75.3)
Others (prebiotic and probiotic, antispasmodics)	56 (37.3)
IV fluids (all the patients admitted in IPD have received IV fluids)	40 (26.7)

**Table 2:** Zinc prescription for the management of under-five diarrhoea by the doctors (N=150)

Zinc prescription for the management of under-five diarrhoea	N (%)
Zinc prescribed as per recommended dose and duration	79 (52.7)
Zinc prescribed with the wrong dose	11 (7.3)
Zinc prescribed with wrong duration	14 (9.3)
Zinc prescribed with wrong dose and duration (both)	9 (6.0)
Zinc was not prescribed	37 (24.7)
Total	150 (100.0)

**Table 3:** Dehydration classification in under-five diarrhoea cases by the doctors (N=150)

Classification of dehydration by doctors	N (%)
Dehydration status was not mentioned in the prescription	134 (89.3)
No dehydration	4 (2.7)
Some dehydration	5 (3.3)
Severe dehydration	1 (0.7)
Dehydration was mentioned but the classification of dehydration was missing	6 (4.0)
Total	150 (100.0)

## Discussion

In our study, most of the under-five diarrhoea cases had received an antibiotic; approximately, two-third had received ORS and half had received recommended dose and duration of zinc. Prebiotics/probiotics/anti-spasmodic were part of more than one-third of the prescriptions. The majority of prescriptions did not classify the dehydration status. The assessment of severe acute malnutrition was not practiced before administering fluid therapy.

ORS with zinc supplementation is found to be effective in childhood diarrhoea [13-15]. In our study, 77.3% of the diarrhoeal disease management prescriptions included the ORS as compared to 58% by Pathak et al. and 22% by Singh et al. from Ujjain and Delhi, respectively [8,16]. The increase in the proportion of ORS use in the prescriptions compared to the previous study may be attributed to the training that was imparted just before the diarrhoeal season in this state. The use of ORS is a cost-effective way to manage diarrhoea in childhood [17]. Promotion of ORS is vital to prevent dehydration and diarrhoea-related under-five mortality. Zinc was prescribed in 75.3% of the prescriptions, however, only 52.7% of the prescriptions had the recommended dose and duration of zinc. This reflects the underutilisation of zinc in the management of childhood diarrhoea. Zinc is useful in reducing the frequency and severity of diarrhoea along with the prevention of future episodes. The efficacy of the diarrhoea management guideline was tested in a randomized controlled trial and it was found to be efficacious [18]. The effectiveness of zinc was also reported in one of the randomized control trials conducted in Bangladesh [19]. Zinc is part and parcel of under-five diarrhoea management. The training of healthcare providers, involved in primary healthcare, is essentially focusing on recommended dose and duration of zinc particularly to prevent the future episode and severity of diarrhoea.

The role of antibiotics in acute childhood diarrhoea is limited to only infectious diarrhoea [20]. Pathak et al. in Ujjain reported 71% of diarrhoea prescriptions contained antibiotics [8]. Our study had similar findings - 80% of the audited prescriptions were having antibiotics. Whereas, Deshmukh et al. in a study conducted in a tertiary care hospital reported 41.3% of prescriptions had antibiotics [17]. The difference in study findings may be due to differences in the study setting. The higher antibiotic prescription in our study reflects the low confidence of the health professionals in ORS and also the lack of appropriate training regarding diarrhoea management. Misuse of antibiotics in diarrhoeal management was reported in Bangladesh [21]. Kotwani et al. also reported irrational use of antibiotics in a study done at primary healthcare in New Delhi [22]. This practice of prescribing antibiotics was also reported by Raghu et al. at Chennai [23]. The excessive and unnecessary use of antibiotics when not indicated will lead to antibiotic resistance and further complications [24].

Assessment of dehydration is key for guiding fluid replacement therapy in diarrhoea. Most of the prescriptions did not mention dehydration status. This may create difficulty in the assessment during referral or discharge. There is a possibility that the child has been examined for dehydration but the dehydration status is not captured in prescription due to busy OPD hours or the healthcare provider has not examined for dehydration status. The second scenario is a much more dangerous situation and creates a problem during referral and discharge. Management of severe dehydration as per plan C is now divided into two parts, where the first part is concerned only about the severe dehydration in normal children and the second part is for the severely malnourished children. Nutritional status assessment in children is essential in case of dehydration management.

The deviation from the management guideline was concerning the use of probiotics and antispasmodics. There are a few reasons which may attribute to the poor adherence to the management guideline. First, drugs like antispasmodics are primarily used for symptomatic pain relief. Second, fever during diarrhoea episodes, satisfying patients’ needs, and insufficient time to explain to patients that antibiotics are not required are the major reasons for a higher antibiotics prescription. Fever creates confusion between viral and bacterial diarrhoea. Third, anti-emetics are prescribed for symptomatic relief of vomiting. But, most of the time parents discontinue rehydration by ORS for vomiting. This is a driving force for the medical practitioner to prescribe unnecessary anti-emetics. Moreover, ORS does not reduce the duration of diarrhoea and that creates a question about the effectiveness of ORS among the parents.

Strength and limitations

This is the first study of its kind in the tribal predominant area of Odisha and the prescription audit was conducted by trained specialists. The representative and adequate sample size of the study enabled us to derive meaningful inferences from the results. The major limitation in the study was that we could not assess whether the prescription was prescribed by AYUSH medical officer or an MBBS medical officer.

## Conclusions

Adherence of doctors to acute diarrhoeal management guidelines for the management of under-five diarrhoea was poor in our study. During the management of acute under-five diarrhoea, most of them had received antibiotics, two-third had received ORS, half had received recommended dose and duration of zinc and prebiotics/probiotics/anti-spasmodic were part of more than one-third of the prescriptions. The majority of prescriptions did not classify the dehydration status and assessment of severe acute malnutrition was not practiced before administering fluid therapy. Further researches and training of healthcare providers are required to improve childhood diarrhoea management in health facilities of tribal areas of India.
